# 

**Published:** 1889-02

**Authors:** 


### CONTENTS:

Article I.—Treatment of Pulpless Teeth. By Frank Hampton Goffe, L. D. S. .......... 433

" II.—The Hands. By Mrs. M. C. Newhaus .......... 439

" III.—Do Benign Tumors Become Malignant? By John B. Harvie, M. D .......... 446

" IV.—Dental Hygiene. By Dr. R. H. Fitch .......... 451

" V.—Leaves From My Experience. By J. A. Robinson. 454

" VI.—Some Suggestions on Metal Cap Crowns. By Mr. W. Mitchell .......... 456

" VII.—A Plea for Tube Teeth. By C. H. Wells, L. D. S.. 459

" VIII.—Fifth, Sixth, Seventh and Eighth Districts Dental Societies of New York State .......... 461

### EDITORIAL.

University of Maryland Dental Department .......... 472

### OBITUARY.

Dr. Thomas Brian Gunning .......... 473

### BIBLIOGRAPHICAL.

The Physicians' Visiting List .......... 474

Vick's Floral Guide .......... 475

Pearson's Dentists' Appointment Book .......... 475

### MONTHLY SUMMARY.

Mistaken Diagnosis .......... 476

The Care of the Nails .......... 476

Hurry is the Cause .......... 477

Food Cranks .......... 477

Who is My Neighbor? .......... 478

Early Rising and Longevity .......... 478

Reflex Pain Following the Extraction of a Tooth .......... 479

Medical Men and Dentists .......... 480

Missionary Dentists .......... 480

